# Global Trends and Research Hotspots of Exercise for Intervening Diabetes: A Bibliometric Analysis

**DOI:** 10.3389/fpubh.2022.902825

**Published:** 2022-07-07

**Authors:** Zhijie Zhang, Yuanchun Zhu, Qingfeng Wang, Tiantian Chang, Chunlong Liu, Yi Zhu, Xueqiang Wang, Xiangyang Cao

**Affiliations:** ^1^Rehabilitation Therapy Center, Luoyang Orthopedic Hospital of Henan Province, Luoyang, China; ^2^Department of Sport Rehabilitation, Shanghai University of Sport, Shanghai, China; ^3^Clinical Medical College of Acupuncture, Moxibustion and Rehabilitation, Guangzhou University of Chinese Medicine, Guangzhou, China; ^4^Department of Musculoskeletal Pain Rehabilitation, The Fifth Affiliated Hospital of Zhengzhou University, Zhengzhou, China

**Keywords:** bibliometric analysis, exercise, diabetes, CiteSpace, research hotspots

## Abstract

**Background:**

Diabetes is a chronic metabolic disease characterized by hyperglycemia that often occurs in adults. Many studies have indicated that exercise is beneficial to the medical management of diabetes. Bibliometric analysis can help investigators to identify the current research concerns to guide future research directions. Nevertheless, the overview bibliometric analysis of this global research topic related to exercise and diabetes is lacking. The present bibliometric study aimed to investigate development trends and research hotspots of exercise and diabetes research and provide researchers with new perspectives in further studies.

**Materials and Methods:**

The articles and reviews regarding exercise and diabetes between 2000 and 2020 were retrieved from the Web of Science Core Collection. The scientometrics analytical tool CiteSpace software was used to analyze the cooperation among countries/institutions/journals/authors, analysis of co-occurrence keywords, keywords bursts, and references.

**Results:**

In all, 3,029 peer-reviewed papers were found with a persistently increased tendency over time. The most prolific country and institution were the USA (965) and Univ Alberta (76), respectively. Diabetes Care published most papers (178) and was the most co-cited journal (2,630). Riddell MC had the most publications (53), and Sigal RJ was the most influential author (503 cited times). Colberg et al.'s paper (co-citation counts: 183) showed the strongest citation bursts by the end of 2020, which was the most representative reference. The four research focuses were mellitus, exercise, physical activity, and glycemic control. The two frontiers trends were sedentary behavior and stress. The combination of aerobic and resistance training can effectively improve glycemic control, decrease HbA1c levels, enhance cardiorespiratory fitness, improve lipid levels, and decrease the demand for non-insulin antihyperglycemic agents.

**Conclusions:**

This study offers a scientific perspective on exercise and diabetes research and provides investigators with valuable information to detect the current research condition, hotspots, and emerging trends for further study.

## Introduction

Diabetes mellitus is a metabolic disease characterized by hyperglycemia for a prolonged period (1). As a chronic medical disease that causes many clinical symptoms and complications, diabetes is related to long-term damage, deterioration, and dysfunction of different organs and systems, especially the kidneys, eyes, nervous system, and cardiovascular system (2). A recent study indicated that about 537 million people worldwide have diabetes, equivalent to 9.8% of the world's adult (20–79 years) population. In 2021, approximately 6.7 million adults aged 20–79 worldwide will die from diabetes, and its complications, with 80% of cases occurring in low- and middle-income countries (3). Given the sizable scope and passive influence of diabetes in the public health area, the effective therapeutic intervention of diabetes has continuously been a research hotspot in medicine.

The previous studies have shown that the problematic situation regarding diabetes could be relieved through advisable medical management and health education (4). With the spread of the idea of “exercise is medicine,” appropriate physical activity and exercise according to the individual characteristics and health status of diabetic patients are crucial for their blood glucose management and overall health (5, 6). Various therapeutic exercise therapies are applied to intervene in diabetes. Many studies have revealed that aerobic exercise, resistance exercise, flexibility, and balance exercises can improve the quality of life of diabetic patients in many aspects, such as enhancing the function of the cardiovascular system, reducing the insulin resistance, improving the blood glucose control, and reducing the risk of falling (7–9). However, there is a lack of relevant quantitative bibliometric analysis summarizing the research progress of exercise therapy intervention in diabetes in recent decades.

Bibliometric analysis, a series of mathematics and statistical analysis for assessing and quantifying publication data, has been utilized to detect the vital researchers, institutions, and countries and their cooperation. Analysis of keywords, references, and co-citation analysis can reveal the global trends and research hotspots (10). In recent years, bibliometrics have been applied in various research fields, such as pain, dyslexia, depression, and cancer rehabilitation (11–13). Outcomes from the bibliometric study can help investigators to identify the current research concerns to guide future research directions. Given the considerable superiority of this method, applying bibliometrics has great significance in the domain of exercise and diabetes research.

This study conducts bibliometrics of exercise and diabetes research based on relevant papers published from 2000 to 2020 to identify the emerging global trends and obtain new insights to guide future research directions.

## Materials and Methods

### Data Acquisition

We performed a comprehensive search of original articles and reviews from 1 January 2000 to 31 December 2020, using the SCIE of the Web of Science Core Collection (WoSCC) on 6 November 2021. Despite the many literature databases available for the study of global trends and research hotspots, we selected the WoSCC database because it included much documents information, especially in the fields of diabetes and public health. The data search strategy was “TI = (exercise^*^ OR kinesitherapy OR training OR “physical activit^*^” OR sport^*^ OR fitness OR walk^*^ OR run^*^ OR swim^*^ OR jog^*^ OR cycling OR pilates^*^ OR yoga OR qigong OR “tai chi”) AND TI = (diabetes OR “diabetes mellitus”) AND Language = English.” In this study, the publication types were restricted to “article” or “review,” time span = 2000–2020.

### Analysis Tools

The CiteSpace 5.8.R 2 Version (Drexel University, Philadelphia, USA), a Java-based application, was utilized to perform the bibliometric analysis on the papers, involving the analysis of publications, journals, authors, references, countries, institutions, and keywords. The CiteSpace V software, an exemplary scientometric analysis method, was utilized to conduct literature analysis (14). The arguments of CiteSpace software were set as follows: time span = 2000–2020, years per slice = 1, term source = all selection, node type = select the corresponding analysis target at a time, selection criteria = top 50, and pruning = none.

In the network map, the different nodes represent a journal, institution, or country, while the magnitude of the node circle represents productivity (15). The links between different nodes reflected the network of collaboration, co-citation, or co-occurrence relationships, and a wider line represents a stronger association (12). The CiteSpace 5.8.R 2 version could identify the citation bursts of references and keywords. A citation burst shows growing attention to the potential research over a specific duration, which is a pivotal indicator for exploring emerging tendencies (16). Furthermore, the Microsoft Excel 2019 software was used to describe the global output and development trend of related papers, and IBM SPSS Statistical 26.0 (SPSS, Inc., Chicago, IL, USA) was applied to execute linear regression analysis to assess the changes in the recent two decades. The *p* < 0.05 was regarded as statistical significance. Finally, according to the Journal Citation Reports (2020), the impact factor (IF) indicates the impact of journals.

## Results

### General Information of Exercise and Diabetes Research

#### Analysis of Publications Outputs

**Figure 1:**
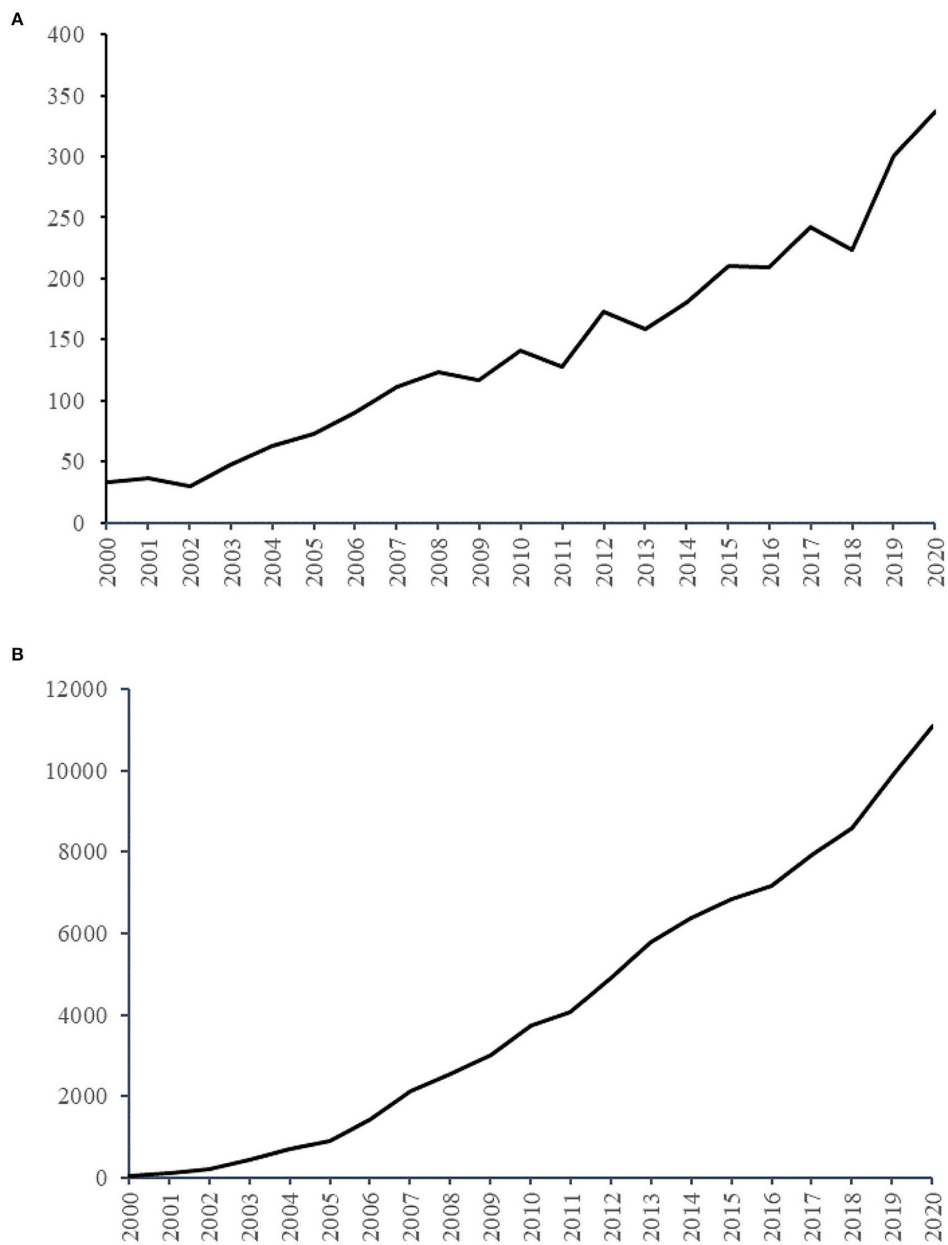
The number of publications and citations. **(A)** The number of annual publications on exercise and diabetes mellitus research from 2000 to 2020. **(B)** The number of annual citations on exercise and diabetes mellitus research from 2000 to 2020.

A total of 3,029 publications from 2000 to 2020 related to exercise and diabetes research, including 2,673 articles and 356 reviews, with an average annual output of 144. As presented in Figure 1A, the annual number of publications rised from 33 in 2000 to 337 in 2020. Although the number of published papers fluctuated in certain periods, the total annual output showed an upward tendency. In addition, the outcomes of linear regression analysis revealed a significant papers growth in the past two decades (*t* = 17. 848, *p* < 0. 001). The 3,029 publications have been cited 96,317 times from 2000 to 2020. As presented in Figure 1B, the amount of citations increased from 26 in 2000 to 11,105 in 2020, and the growth was statistically significant (*t* = 23.958, *p* < 0.001).

#### Analysis of Countries and Institutions

Eighty-one countries and 3,783 institutions published 3,029 publications on exercise and diabetes research. The list of the top 10 countries and institutions is presented in Table 1.

**Table 1:** Top 10 countries and institutions on exercise and diabetes mellitus research from 2000 to 2020.

**Rank**	**Country**	**Publications**	**Centrality**	**Institution**	**Publications**	**Centrality**
1	USA	965	0.57	Univ Alberta (Canada)	76	0.05
2	Canada	347	0.07	Univ Copenhagen (Denmark)	67	0.10
3	England	305	0.33	York Univ (Canada)	60	0.09
4	Australia	287	0.15	Univ Calgary (Canada)	52	0.04
5	Peoples R China	148	0.01	Univ Queensland (Australia)	51	0.07
6	Brazil	146	0.07	Univ Colorado (USA)	48	0.08
7	Italy	128	0.07	Harvard Univ (USA)	42	0.10
8	Japan	107	0.04	Univ Sydney (Australia)	42	0.06
9	Germany	105	0.04	Maastricht Univ (Netherlands)	39	0.05
10	Denmark	104	0.04	Univ Pittsburgh (USA)	37	0.04

**Figure 2:**
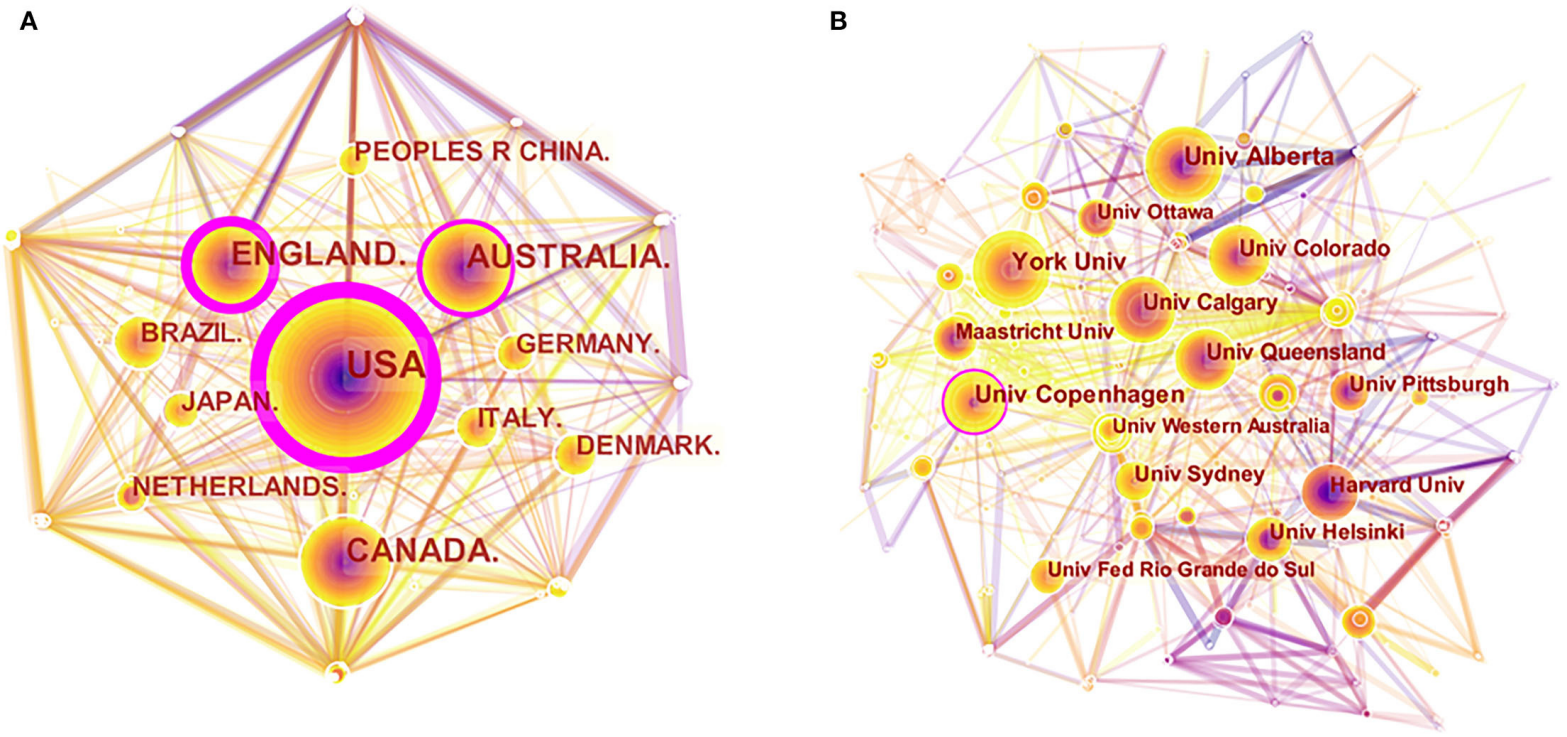
The cooperation of countries and institutions. **(A)** Map of countries with publications in exercise and diabetes mellitus research from 2000 to 2020. **(B)** Map of institutions with publications in exercise and diabetes mellitus research from 2000 to 2020.

As presented in Figure 2A, the cooperation network map of countries was produced by the CiteSpace software. The purple ring reflects centrality, and the country with high centrality was known as a vital point in the publications. The USA had the most publications (965 publications), followed by Canada (347 publications), England (305 publications), Australia (287 publications), and China (148 publications). The USA also had the highest centrality (0.57), followed by England (0.33), Australia (0.15), Canada (0.07), and Brazil (0.07).

The collaboration network map of institutions is shown in Figure 2B. Alberta University had the most studies (76 publications), followed by the University of Copenhagen (67 publications), York University (60 publications), University of Calgary (52 publications), and University of Queensland (51 publications). Copenhagen and Harvard University showed the highest centrality (0.10).

#### Analysis of Journals and Co-Cited Journals

In all, 688 journals published papers on exercise and diabetes research. The top 10 journals generated 775 documents, making up 25.34% of the 3,029 publications. In terms of the number of published papers on exercise and diabetes research, as presented in Table 2, Diabetes Care (IF 2020 = 19.112) was the most productive journal (178 publications, 5.88%), followed by the Diabetologia (96 publications), Diabetes Research and Clinical Practice (87 publications), and Diabetic Medicine (81 publications). Among the top 10 journals, the IF was ranked between 2.140 and 19.112, the average IF was 6.516.

**Table 2:** Top 10 journals related to exercise and diabetes mellitus research from 2000 to 2020.

**Rank**	**Journal**	**Publications**	**Percentage (%)**	IF^*^(2020)
1	Diabetes Care	178	5.88%	19.112
2	Diabetologia	96	3.17%	10.122
3	Diabetes Research and Clinical Practice	87	2.87%	5.602
4	Diabetic Medicine	81	2.67%	4.359
5	Plos One	76	2.51%	3.240
6	Medicine and Science in Sports and Exercise	62	2.05%	5.411
7	Canadian Journal of Diabetes	56	1.85%	4.190
8	Diabetes Educator	49	1.62%	2.140
9	Pediatric Diabetes	47	1.55%	4.866
10	Diabetes Technology & Therapeutics	43	1.42%	6.118

* *IF according to Journal Citation Reports (2020). IF, impact factor*.

**Figure 3:**
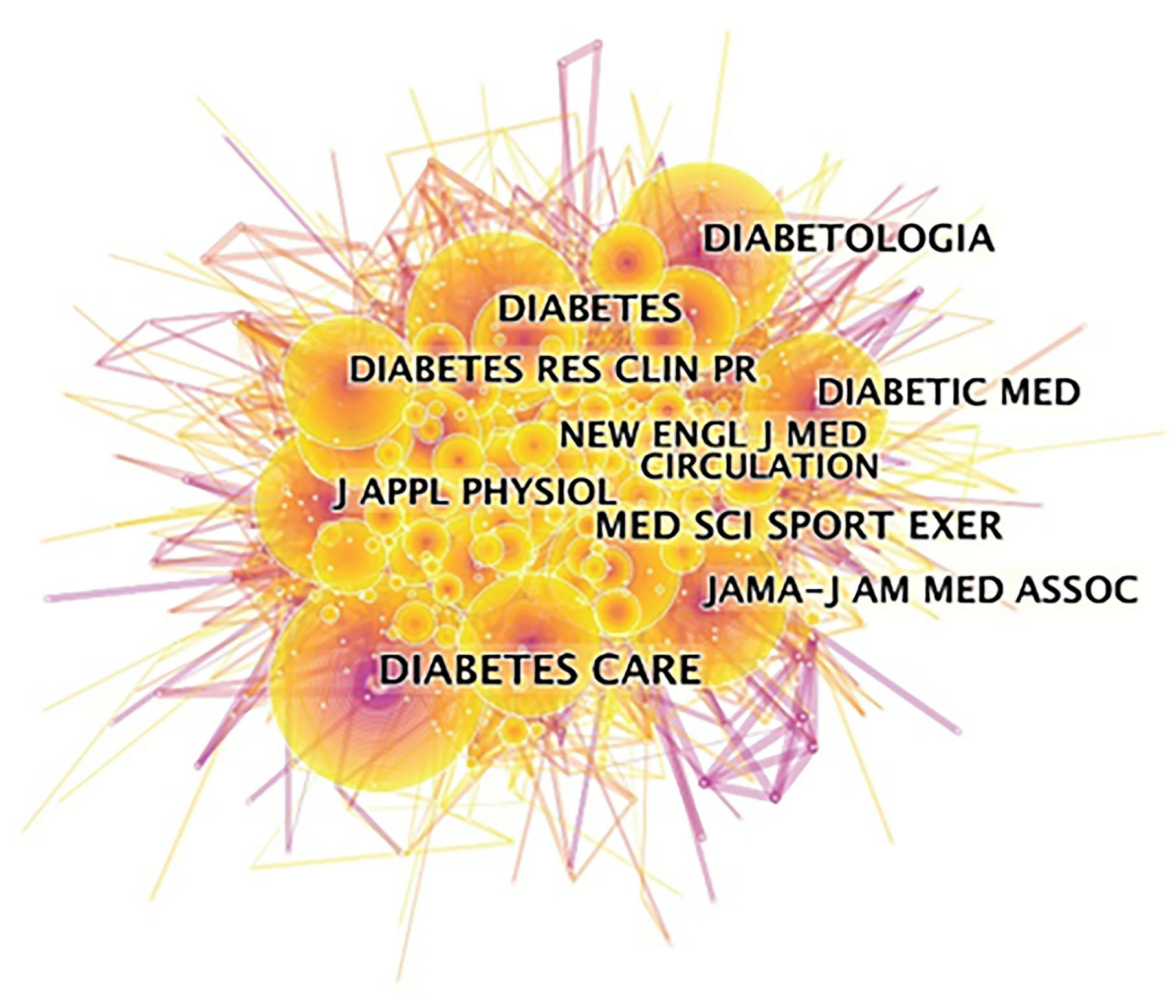
Journal co-citation map associated with publications on exercise and diabetes mellitus research from 2000 to 2020.

In addition, Figure 3 presented the scholarly journals co-cited map associated with publications on exercise and diabetes research. Figure 3 and Table 3 showed co-citation combined with centrality. The top 5 co-cited journals were Diabetes Care, Diabetologia, Medicine and Science in Sports and Exercise, Diabetes, and JAMA-Journal of the American Medical Association.

**Table 3:** Top 10 co-cited journals on exercise and diabetes mellitus research from 2000 to 2020.

**Rank**	**Co-citation counts**	**Cited journal**	**Centrality**	**Cited journal**
1	2,630	Diabetes Care	0.04	British Journal of Nutrition
2	1,599	Diabetologia	0.04	Journal of the American Dietetic Association
3	1,576	Medicine and Science in Sports and Exercise	0.04	Journal of Hypertension
4	1,369	Diabetes	0.03	Annals of Medicine
5	1,294	JAMA-Journal of the American Medical Association	0.03	Biochemical and Biophysical Research Communications
6	1,155	New England Journal of Medicine	0.03	Endocrinology
7	1,081	Journal of Applied Physiology	0.03	Patient Education and Counseling
8	983	Diabetes Research and Clinical Practice	0.03	Journals of Gerontology Series A- Biological Sciences and Medical Sciences
9	975	Diabetic Medicine	0.03	American Journal of Public Health
10	901	Circulation	0.03	American Journal of Physiology-Heart and Circulatory Physiology

#### Analysis of Authors

**Figure 4:**
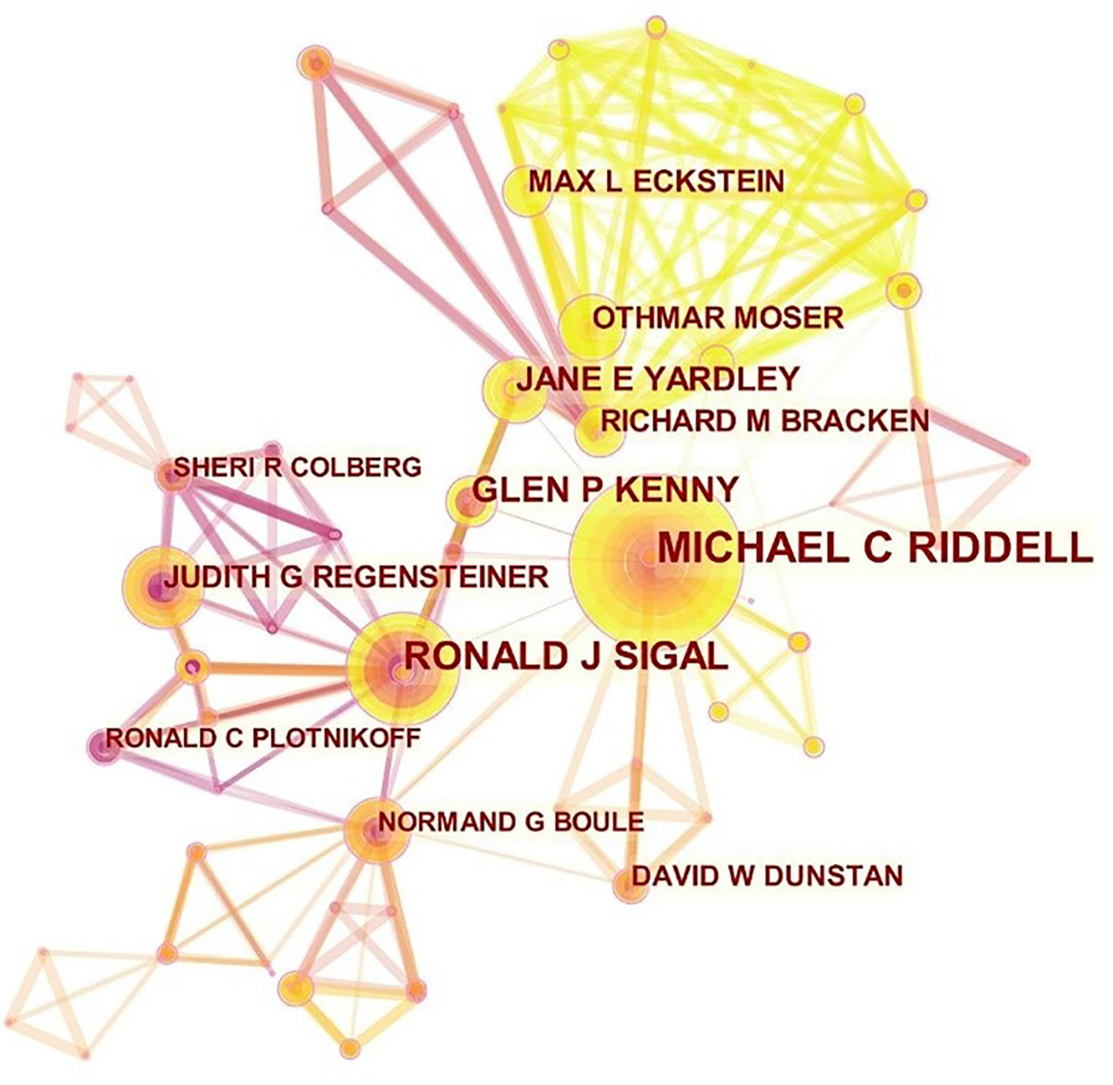
Network map of authors who contributed to exercise and diabetes mellitus research from 2000 to 2020.

As many as 13,000 authors published 3,029 papers contributing to research on exercise and diabetes. To summarize the cooperation among authors, a network map was generated using CiteSpace (Figure 4). Judging from the situations of cooperation map, the scale of collaboration among authors was relatively light and needed to strengthen the overall connection. The top 10 authors, co-cited authors of exercise and diabetes research, are presented in Table 4. Among the top 10 authors, Riddell MC ranked first with 53 articles, followed by Sigal RJ with 39 articles, Kenny GP with 24 articles, and Yardley JE with 22 articles. Riddell MC came from York University, Canada, Sigal RJ, Kenny GP, and Yardley JE were from the University of Ottawa, Canada, Bracken RM, and Moser O came from Swansea University, United Kingdom, Regensteiner JG came from University of Colorado, USA, Eckstein ML came from the Division of Endocrinology and Diabetology, Medical Austria, Blair SN came from the University of South Carolina, USA, and Plotnikoff RC came from the University of Alberta, Canada.

**Table 4:** Top 10 authors and co-cited authors on exercise and diabetes mellitus research from 2000 to 2020.

**Rank**	**Author**	**Published articles**	**Co-cited author**	**Cited times**	**Centrality**
1	Riddell MC	53	Sigal RJ	503	0.02
2	Sigal RJ	39	Colberg SR	464	0.03
3	Kenny GP	24	Boule NG	405	0.08
4	Yardley JE	22	Amer Diabet Assoc	332	0.02
5	Bracken RM	18	Knowler WC	304	0.01
6	Regensteiner JG	17	Church TS	253	0.04
7	Moser O	16	Balducci S	226	0.03
8	Eckstein ML	15	Dunstan DW	224	0.04
9	Blair SN	14	Hu FB	217	0.03
10	Plotnikoff RC	14	Umpierre D	204	0.01

### Research Hotspots

#### Analysis of Co-Cited Authors

**Figure 5:**
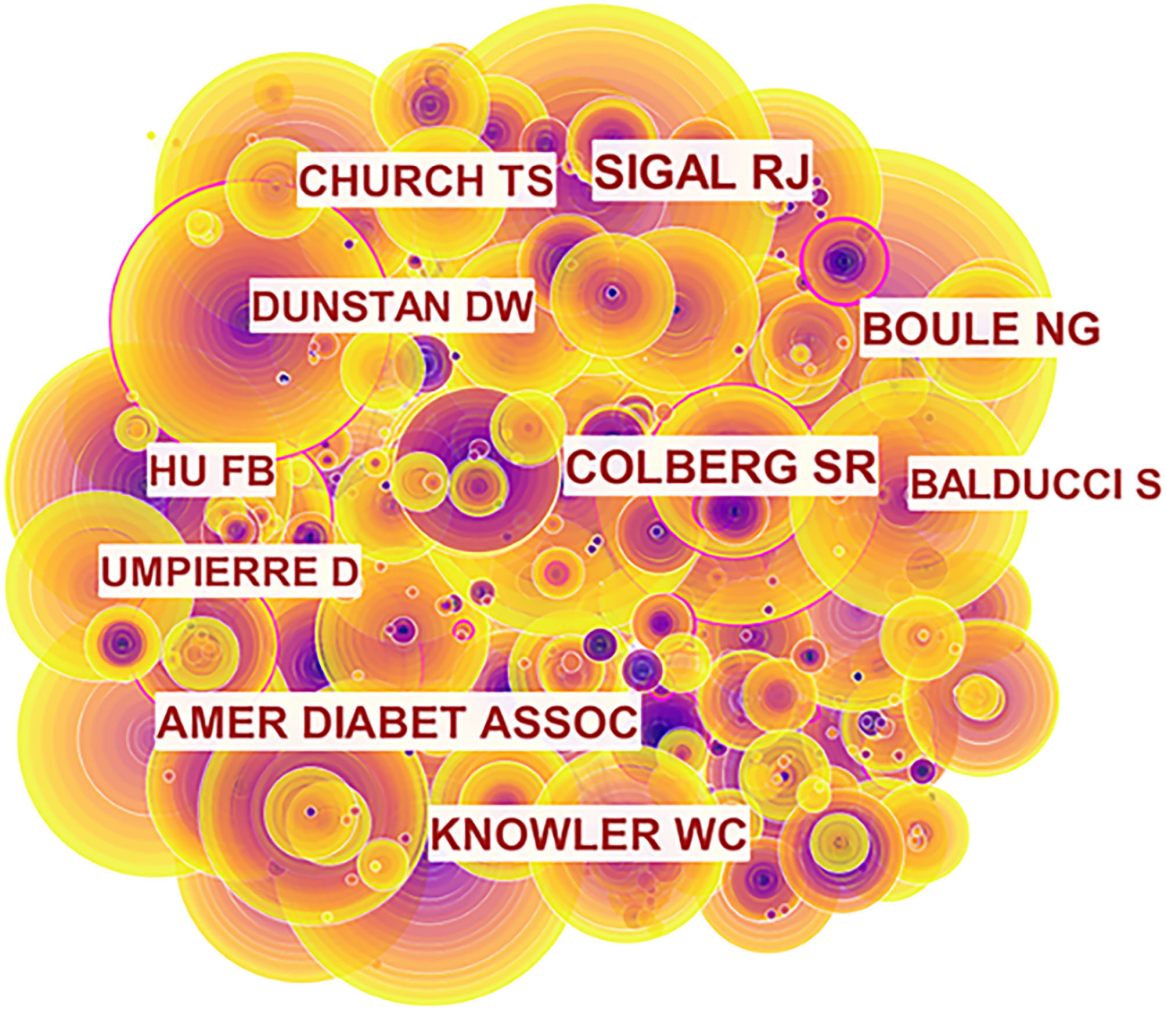
Map of co-cited authors related to exercise and diabetes mellitus research from 2000 to 2020.

The network map of co-cited authors is shown in Figure 5. Sigal RJ ranked first with 503 cited times, followed by Colberg SR, Boule NG, and Amer Diabet Assoc with 464, 405, and 332 cited times, respectively (Table 4). Among them, Boule NG had the highest centrality (0.08). The top 10 co-cited authors' citation amounts exceeded 200, which indicated that they were yauld and influential researchers in the domain of exercise and diabetes research. Sigal RJ came from the muscle health research center at York University, Canada. Colberg SR came from the department of human movement sciences at Old Dominion University, USA. Boule NG was from the institute of human kinetics at the University of Ottawa, Canada. Knowler WC was from the diabetes prevention program coordinating center at George Washington University, USA.

#### Analysis of Co-Occurrence Keywords

**Figure 6:**
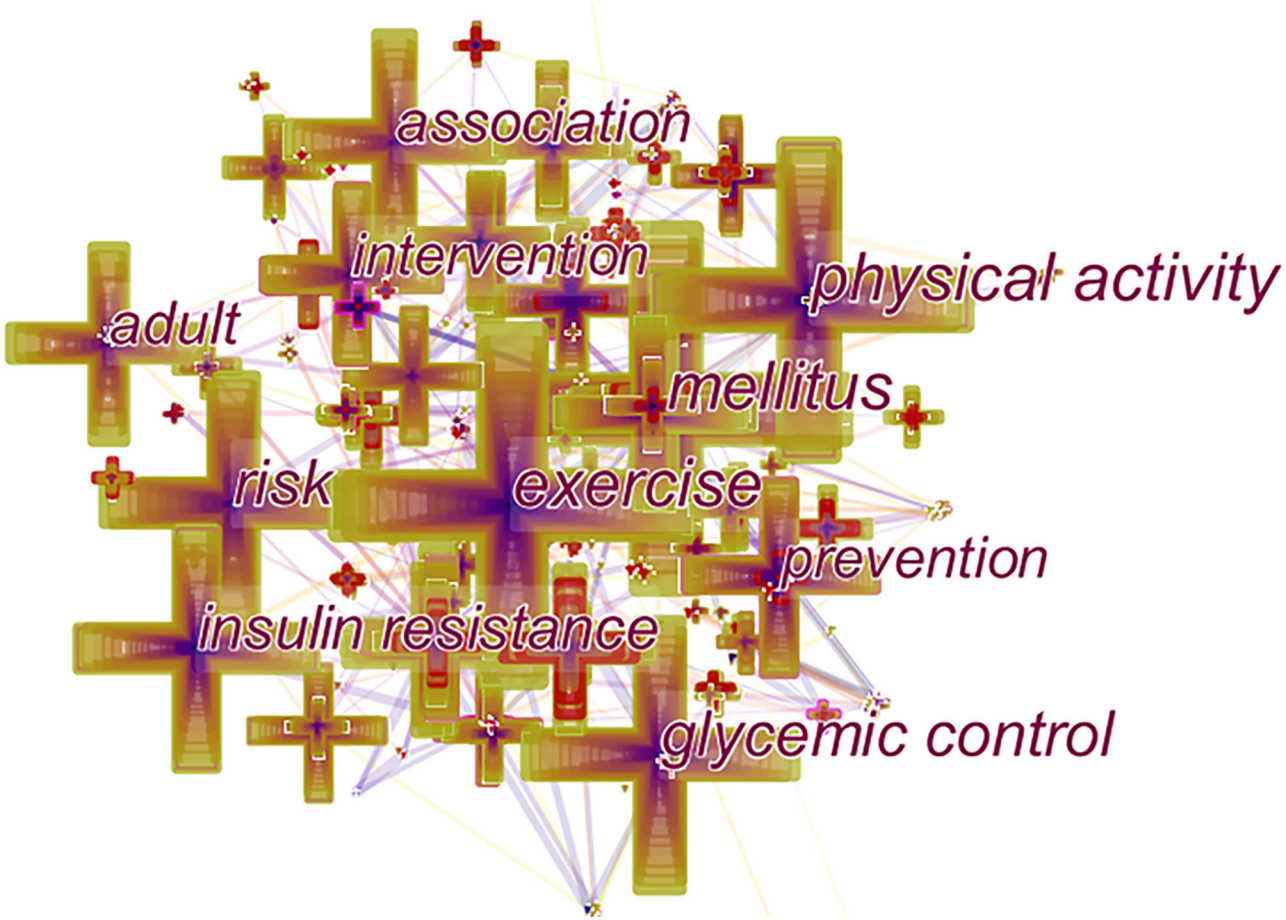
Map of the co-occurrence keywords in publications on exercise and diabetes mellitus research from 2000 to 2020.

Figure 6 shows the co-occurrence network of keywords from 2000 to 2020, indicating the scope of research topics on exercise and diabetes. As shown in Table 5, The most common keyword was “mellitus,” with 637 co-occurrences, followed by “exercise” (591 times), “physical activity” (556 times), “glycemic control” (406 times), “risk” (382 times), “insulin resistance,” “association,” “adult,” “prevention,” and “intervention.” Among them, “intervention” had the highest centrality (0.28).”

**Table 5:** Top 10 keywords related to exercise and diabetes mellitus research from 2000 to 2020.

**Rank**	**Frequency**	**Keyword**	**Centrality**	**Rank**	**Frequency**	**Keyword**	**Centrality**
1	637	Mellitus	0.08	6	302	Insulin resistance	0.15
2	591	Exercise	0.02	7	272	Association	0.02
3	556	Physical activity	0.14	8	262	Adult	0.08
4	406	Glycemic control	0.00	9	218	Prevention	0.23
5	382	Risk	0.02	10	216	Intervention	0.28

#### Analysis of Co-Cited References

In all, 67,063 references were performed from 3,029 papers to analyze co-cited references. Table 6 lists the top 5 co-cited references related to exercise and diabetes research between 2000 and 2020. The first article was the study of Colberg et al. (5) had the maximum co-citation counts (183), which proposed that physical activity/exercise should be a part of glycemic control and overall health management for all patients with diabetes. And the specific type and intensity of exercise will vary depending on the type of diabetic patient, age, and presence of diabetes-related health complications.

**Table 6:** Top 5 co-cited references related to exercise and diabetes mellitus research from 2000 to 2020.

**Rank**	**Co-citation counts**	**Co-cited reference**	**References**
1	183	Physical Activity/Exercise and Diabetes: A Position Statement of the American Diabetes Association	(5)
2	158	Physical activity advice only or structured exercise training and association with HbA1c levels in type 2 diabetes: a systematic review and meta-analysis	(17)
3	150	Physical Activity/Exercise and Type 2 Diabetes A consensus statement from the American Diabetes Association	(18)
4	125	Reduction in the incidence of type 2 diabetes with lifestyle intervention or metformin	(19)
5	124	Effects of aerobic training, resistance training, or both on glycemic control in type 2 diabetes: a randomized trial	(20)

The second article was the study of Umpierre et al. (17) with the co-citation counts of 158, which reviewed that structured exercise training was related to improved glycemic control in persons with type 2 diabetes. Still, physical activity was only related to improved glycemic control when combined with dietary recommendations.

The third article was the study of Sigal et al. (18) with the co-citation counts of 150, a consensus statement from the American Diabetes Association. They summarized the exercise/physical activity associated with diabetes and made the different levels of the recommendations based on the levels of evidence.

The fourth and fifth articles were published in 2002 and 2007 by Knowler et al. (19) and Sigal et al. (20), respectively. Knowler et al. (19) found that lifestyle intervention and metformin treatment delayed and prevented type 2 diabetes, particularly in the former. Sigal et al. (20) discovered that aerobic and resistance training improved glycemic control and combining the two methods was more effective than either one alone.

### Global Trends of Exercise and Diabetes Research

#### Analysis of Keywords with the Strongest Citation Bursts

The top 45 keywords with the strongest citation bursts on exercise and diabetes study between 2000 and 2020 are presented in Table 7. The latest burst keywords were “system,” “type 1,” “sedentary behavior,” “stress”.

**Table 7:** Top 45 keywords with the strongest citation bursts.

**Keywords**	**Year**	**Strength**	**Begin**	**End**	**2000–2020**
NIDDM	2000	17.35	2000	2007	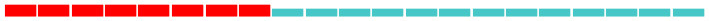
Impaired glucose tolerance	2000	13.57	2000	2010	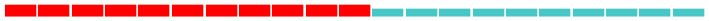
Insulin	2000	13.32	2000	2007	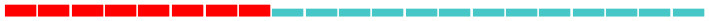
Women	2000	6.96	2000	2007	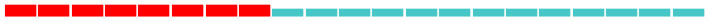
Human	2000	3.9	2000	2008	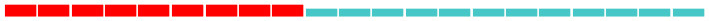
IDDM	2000	3.6	2000	2007	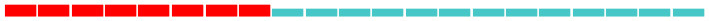
Metabolism	2000	3.4	2000	2003	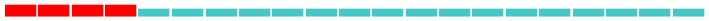
Men	2000	10.54	2001	2007	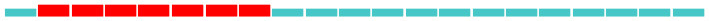
Glucose tolerance	2000	8.18	2001	2006	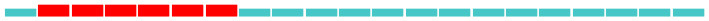
Glucose transport	2000	5.39	2001	2009	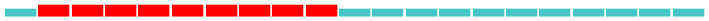
Blood pressure	2000	4.53	2001	2005	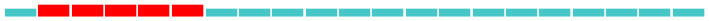
Life style	2000	13.49	2002	2007	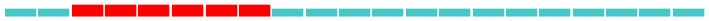
Coronary heart disease	2000	6.65	2002	2007	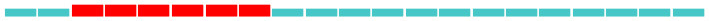
Prevention	2000	6.11	2002	2007	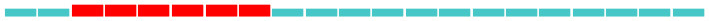
3rd national health	2000	3.66	2002	2005	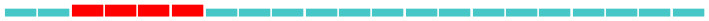
Rat	2000	3.58	2002	2008	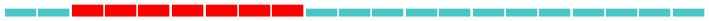
Gene expression	2000	5.26	2003	2011	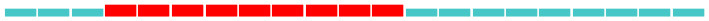
Response	2000	3.63	2003	2008	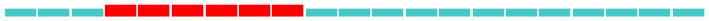
Tolerance	2000	5.96	2004	2013	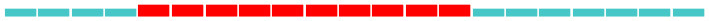
Homeostasis model assessment	2000	5.47	2004	2008	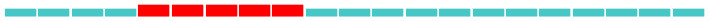
Coronary artery disease	2000	4.19	2004	2013	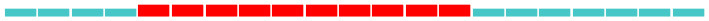
Determinant	2000	6.82	2005	2010	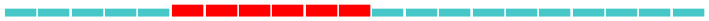
Necrosis factor alpha	2000	5.09	2005	2008	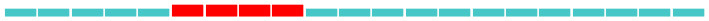
Cardiomyopathy	2000	3.46	2005	2009	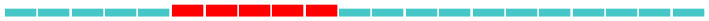
Counterregulatory response	2000	5.09	2006	2010	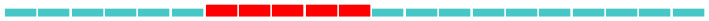
Improves glycemic control	2000	6.9	2007	2010	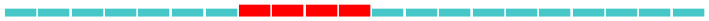
Plasma glucose	2000	3.82	2007	2010	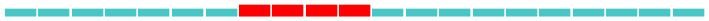
Predictor	2000	3.65	2007	2010	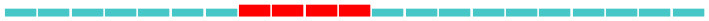
Self management	2000	4.8	2008	2012	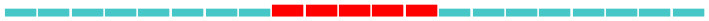
Atherosclerosis	2000	3.3	2008	2013	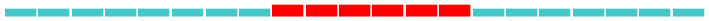
Reduction	2000	6.08	2009	2016	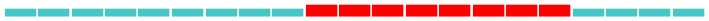
Energy expenditure	2000	3.19	2009	2013	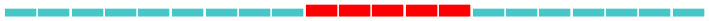
Hypertension	2000	3.6	2010	2013	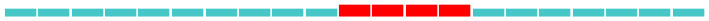
Randomized controlled trial	2000	7.95	2011	2018	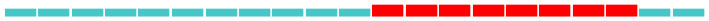
US adult	2000	4.47	2011	2017	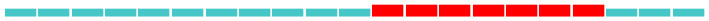
Validity	2000	3.9	2011	2016	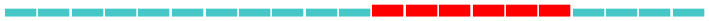
American college	2000	3.32	2011	2016	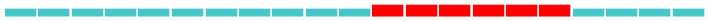
Cardiovascular risk factor	2000	7.23	2012	2015	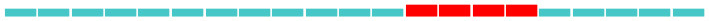
Endothelial function	2000	4.72	2012	2015	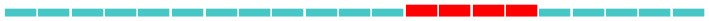
MA	2000	3.14	2014	2017	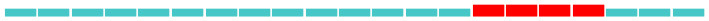
Gait	2000	3.77	2015	2018	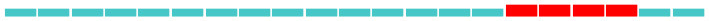
System	2000	4.41	2016	2020	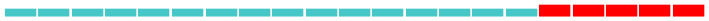
Type 1	2000	3.99	2016	2020	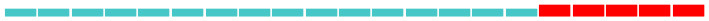
Sedentary behavior	2000	3.63	2016	2020	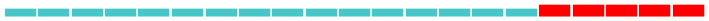
Stress	2000	3.54	2016	2020	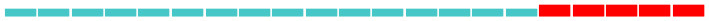

#### Analysis of References with the Strongest Citation Bursts

The top 20 references from 2000 to 2020 are presented in Table 8. The red rectangles indicate references cited continually, and the other color rectangles were cited infrequently. Analysis of the citation bursts of references, the papers between 2014 and 2017 initiated the research upsurge of exercise therapy for diabetes. The authors of the top five strongest citation bursts references were Colberg SR, Riddell MC, Mitranun W, Bohn B, and Jelleyman C. The highest strength burst reference started in 2016 from Colberg et al. (5).

**Table 8:** Top 20 references co-citation with the strongest citation bursts.

**References**	**Year**	**Strength**	**Begin**	**End**	**2000–2020**
Colberg SR, 2016, DIABETES CARE, V39, P2065, DOI 10.2337/dc16-1728	2016	66.65	2018	2020	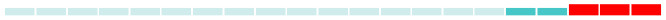
Riddell MC, 2017, LANCET DIABETES ENDO, V5, P377, DOI 10.1016/S2213-8587(17)30014-1	2017	39	2018	2020	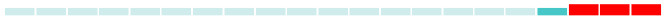
Mitranun W, 2014, SCAND J MED SCI SPOR, V24, P0, DOI 10.1111/sms.12112	2014	18.91	2016	2020	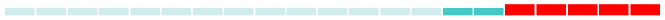
Bohn B, 2015, DIABETES CARE, V38, P1536, DOI 10.2337/dc15-0030	2015	17.09	2017	2020	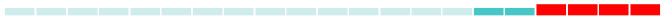
Jelleyman C, 2015, OBES REV, V16, P942, DOI 10.1111/obr.12317	2015	12.8	2017	2020	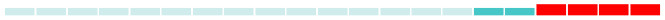
Moser O, 2015, PLOS ONE, V10, P0, DOI 10.1371/journal.pone.0136489	2015	11.37	2017	2020	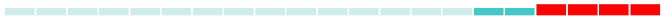
WHO, 2016, GLOBAL REPORT DIABET, V0, P0	2016	11.01	2017	2020	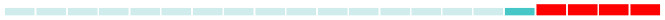
Hollekim-Strand SM, 2014, J AM COLL CARDIOL, V64, P1758, DOI 10.1016/j.jacc.2014.07.971	2014	10.61	2016	2020	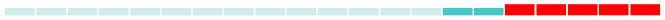
Aune D, 2015, EUR J EPIDEMIOL, V30, P529, DOI 10.1007/s10654-015-0056-z	2015	10.56	2016	2020	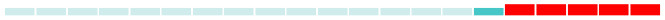
Weston KS, 2014, BRIT J SPORT MED, V48, P1227, DOI 10.1136/bjsports-2013-092576	2014	10.22	2016	2020	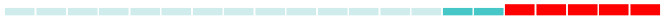
Quirk H, 2014, DIABETIC MED, V31, P1163, DOI 10.1111/dme.12531	2014	10.1	2015	2020	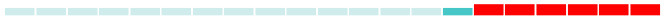
McAuley SA, 2016, DIABETOLOGIA, V59, P1636, DOI 10.1007/s00125-016-3981-9	2016	9.94	2017	2020	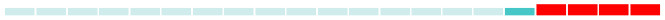
Schwingshackl L, 2014, DIABETOLOGIA, V57, P1789, DOI 10.1007/s00125-014-3303-z	2014	9.82	2016	2020	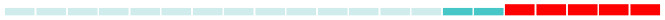
Grace A, 2017, CARDIOVASC DIABETOL, V16, P0, DOI 10.1186/s12933-017-0518-6	2017	9.12	2018	2020	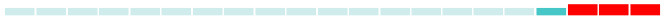
Robertson K, 2014, PEDIATR DIABETES, V15, P203, DOI 10.1111/pedi.12176	2014	8.64	2016	2020	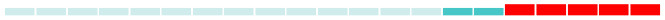
Lascar N, 2014, PLOS ONE, V9, P0, DOI 10.1371/journal.pone.0108019	2014	8.53	2018	2020	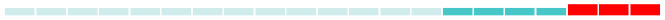
AmericanDiabetesAssociation, 2015, Diabetes Care, V38 Suppl, P0, DOI 10.2337/dc15-S005	2015	8.35	2017	2020	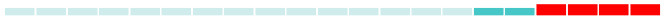
Franc S, 2015, DIABETES OBES METAB, V17, P1150, DOI 10.1111/dom.12552	2015	8.16	2017	2020	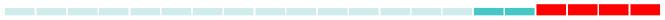
Campbell MD, 2015, BMJ OPEN DIAB RES CA, V3, P0	2015	7.93	2016	2020	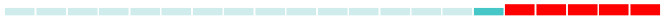
Moser O, 2016, NUTRIENTS, V8, P0, DOI 10.3390/nu8080489	2016	7.81	2018	2020	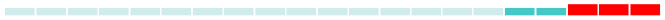

## Discussion

### General Information for Exercise and Diabetes Research

The research on exercise and diabetes has received tremendous attention in the past two decades. This phenomenon may be related to the increase in the morbidity of diabetes and the increased economic burden it brings, and the popularization of the concept of “exercise is medicine.” Moreover, eight are developed countries among the top 10 countries, and only two are developing countries. There was still a considerable gap between developing and developed countries in this research domain. Moreover, the USA has the most published papers (965) and the highest centrality (0.57), which shows that the USA is the leading in this research area. The output institutions are mainly from Univ Alberta and Univ Calgary of Canada, Univ Copenhagen of Denmark, York Univ of USA, and Univ Queensland of Australia. The top 10 institutions make up 16.969% of total publication yields, which implied that they had obtained considerable academic achievements. Nevertheless, compared with the collaboration between countries, the collaboration between those institutions was not apparent. Cooperation helps researchers who investigate exercise and diabetes research exchange thoughts and resources, critical for further research development. Thus, stronger cooperation relationships should be built among researchers, countries, and institutions.

The articles about exercise and diabetes research were published in 688 diverse academic periodicals, and the top 10 journals came out 25.34% of publications. Among the top 10 journals with IF > 10 (Diabetes Care, IF, 2020 = 19.112 and Diabetologia IF, 2020 = 10.122), and three had 5 < IF < 10. About 23.969% (IF, 2020 > 5.000, 15.385%; 3.000 ≤ IF, 2020 ≤ 5.000, 8.584%) of the 3,029 publications were came out in the top 10 periodicals with high IF (>3.000). Thus, it was challenging to publish relevant publications in high impact factor journals.

The data on crucial authors can help investigators to find the underlying cooperators. Authors Riddell MC, Sigal RJ, Colberg SR, and Boule NG were the most productive and influential authors in the exercise and diabetes area, as identified by an omnibus analysis of amounts of published papers and co-citations. Riddell MC offered the latest consensus on exercise management for type 1 diabetic patients who exercise regularly, including blood glucose goals when performing different exercise programs (21). Sigal et al. summarized the up-to-date clinical research progress related to exercise and type 2 diabetes and recommendations (18). Colberg et al. provided a review and evidence-based recommendations concerning exercise in individuals with diabetes and prediabetes (5). Boule et al. showed that regular exercise has a significant clinical effect on maximal oxygen uptake in individuals with type 2 diabetes and higher intensity exercise has additional benefits on cardiorespiratory fitness (22).

### Research Hotspots for Exercise and Diabetes Research

The research focuses of the current study were explored from the three sides of co-occurrence keywords, co-cited authors, and co-cited references, which refer to numerous interrelated topics discussed within a particular scholastic discipline (10).

According to the co-cited authors, Sigal et al., Colberg et al., and Boule et al. had a long-term collaboration (5, 20, 22, 23). Sigal RJ and Colberg SR often cooperated to give recommendations and precautions for different types of exercises to persons with type 2 diabetes, such as the types and quantities of exercise that can be performed by individuals with type 2 diabetes, along with the announcements required to maximize the safety of physical activities in those using multifarious medications and accompany with diabetes-related complications. Boule NG mainly focused on applying aerobic exercise, resistance exercise, and mixed exercise mode in patients with type 2 diabetes, which can effectively improve glycemic control, enhance cardiorespiratory fitness, and decrease the demand for non-insulin antihyperglycemic agents.

Our analysis of co-occurrence keywords indicated that the four most commonly used keywords are mellitus, exercise, physical activity, and glycemic control. Exercise is a crucial component of diabetes management because it can decrease blood glucose levels, lose weight, and improve lipid levels (23). However, a systematic review and meta-analysis about the influences of structured exercise programs (aerobic, resistance, or both) on HbA1c (A1C) and body weight in individuals with type 2 diabetes revealed that A1C decreased remarkably after exercise intervention. On the contrary, there was no significant change in body mass after intervention (24). Hence, structured exercise regimens had a significant beneficial impact on A1C and glycemic control, and this influence was not primarily regulated by bodyweight reduction. Exercise more than 2.5 h per week is related to greater A1C decline than those who do not have enough structured exercise time (17). Moreover, the combination of aerobic and resistance training can improve the level of A1C in patients with type 2 diabetes, but this beneficial effect cannot be achieved by aerobic or resistance training alone (20). Although exercise has long been considered the sustentaculum of diabetes management, many physicians have not prescribed it. According to the five references cited most, physicians should be reminded that managements used to intervene in type 2 diabetes should aggrandize lifestyle improvements, such as increased physical activity level in daily life, rather than replace them (23).

### Global Trends of Exercise and Diabetes Research

Keywords with citation bursts are known as indicators of frontier topics. Two emerging trends in exercise and diabetes research were identified in line with the comprehensive analysis of the latest keyword bursts; details are listed as follows:

- Sedentary behavior: Sedentary behaviors refer to activities that have a low energy expenditure (≤1.5 metabolic equivalents) while in a sitting or lying down position (25). Greater sedentary behavior time was positively related to an increased risk for premature mortality and type 2 diabetes incidence (26). Conducted a cohort study among adults over 45 years old in Australia, the results showed that subjects who reported <8 h of sedentary time per day could decrease 14% the risk of latently preventable hospitalization. Moreover, the deleterious effects of sedentary behavior time usually decreased in magnitude among persons who performed higher physical activity/exercise levels than lower levels (27). Recent studies found that breaking up the sedentary behavior may be related to beneficial changes in the cardiometabolic and inflammatory risk in the type 2 diabetes population, interrupting sedentary behavior time by adding low-intensity physical activity breaks (28, 29). Greater evidence indicated that interrupting prolonged sedentary status by light-intensity walking is an effective method for improving postprandial glucose level (30), and performing light ambulation breaks reduced the 7 h glucose of type 2 diabetes patients compared with the prolonged sitting position (31). However, physical activity/exercise strategies to reduce the adverse effects of sedentary behavior on healthy populations and diabetic patients should be further investigated.
- Stress: The COVID-19 pandemic is a severe medical crisis that has had a powerful negative impact on daily life worldwide. In order to control the epidemic, many countries implemented lockdown strategies that led to considerable changes in social behavior (32). A previous study revealed a lockdown related to increased levels of emotional distress (33). These alterations in daily life, behavior patterns, physical activities, and increased emotion of stress and anxiety are all known to affect the healthcare and glycemic control of diabetes patients (34, 35). Moreover, chronic psychological stress has been associated with high level of A1C (36). The previous studies of diabetes found that acute psychological stress caused a delayed reduction in the postprandial glucose concentrations (35, 37). However, the potential pathomechanism for the different glucose concentrations in response to psychological stress remains unclear. Thus, diabetes healthcare professionals should consider the aspects of psychological stress alteration when discussing diabetes management and exercise promotes health.

Instead of exploring all the burst references, the subsequent discussions will concern the top 5 references by the end of 2020.

As presented in Table 8, a study by Colberg et al. (5) represented the strongest burst by the end of 2020. This research offered a review and evidence-based recommendations about exercise/physical activity in individuals with prediabetes, type 1, type 2, and gestational diabetes. Exercise should be prescribed to all persons with diabetes to manage glycemic control and healthcare. Adults should be encouraged to reduce the sedentary time and interrupt the sedentary behavior with frequent activity.

The study by Riddell et al. (21) represented the second strongest burst by the end of 2020. This review offered the latest consensus on exercise management for persons with type 1 diabetes mellitus who exercise regularly, involving glucose goals for safe and effective physical activities and nutritional and insulin dose regulations to defend against exercise-related glucose fluctuations.

The following paper investigated the effects of continuous and interval aerobic exercise training on macro- and microvascular reactivity and glycemic control in individuals with type 2 diabetes (38). This study indicated that continuous and interval exercise training effectively improved glycemic control and maximal aerobic fitness. The high-intensity interval exercise training could produce greater improvements in vascular complications that individuals with type 2 diabetes often suffer.

The fourth paper investigated the effects of exercise on glycemic control and cardiovascular risk factors in subjects with type 1 diabetes (39). This research found a negative correlation between physical activity and A1C, dyslipidemia, diabetic ketoacidosis, body mass index, hypertension, retinopathy, and microalbuminuria. Therefore, the authors recommended regular physical activity for patients with type 1 diabetes.

The fifth research explored the impact of high-intensity interval training (HIIT) on insulin resistance and glucose regulation compared to continuous training and control conditions (40). The results showed a significant reduction in insulin resistance after HIIT in comparison to other groups. Compared with control conditions, A1C and body weight decreased significantly following HIIT. HIIT appears to improve metabolic health in those at risk of or with type 2 diabetes.

This study is the first to investigate the global trends and research hotspots of exercise and diabetes research based on papers published between 2000 and 2020 by a bibliometrics method. Our study offers new historical perspectives for scientific growth and analyzes different sides of academic literature. However, this study still has several limitations. First, the relevant papers retrieved were only performed in SCIE of WoS and lacked other databases, such as Scopus. Second, we excluded the non-English publications, and this limitation may cause publication bias.

## Conclusion

This bibliometric study could help researchers to find the publication patterns, research hotspots, and emerging exercise, and diabetes research trends from 2000 to 2020. The current research status indicated that exercise and diabetes research still has promising development potential. The most influential country, institution, journal, and author were the USA, Univ Alberta, Diabetes Care, and Sigal RJ. “Sedentary behavior” and “stress” may be the up-to-date research frontiers. In general, this study offers a scientific perspective on exercise and diabetes study, providing helpful information for relevant investigators, funding agencies, and policymakers.

## Data Availability Statement

The original contributions presented in the study are included in the article/supplementary material, further inquiries can be directed to the corresponding author.

## Author Contributions

YuZ, ZZ, and XC conceptualized, designed, and performed the study. YuZ, QW, and TC contributed to collecting data. YuZ, ZZ, TC, and CL interpreted data and wrote the manuscript. YuZ, CL, YiZ, XW, XC, and ZZ revised the manuscript. All authors approved the final version of the manuscript.

## Funding

This research was supported by the Construction Project of Administration of Traditional Chinese Medicine of Henan Province (2021-30) and the Project of Science Research of Traditional Chinese Medicine of Henan Province of China (2019ZY1028).

## Conflict of Interest

The authors declare that the research was conducted in the absence of any commercial or financial relationships that could be construed as a potential conflict of interest.

## Publisher's Note

All claims expressed in this article are solely those of the authors and do not necessarily represent those of their affiliated organizations, or those of the publisher, the editors and the reviewers. Any product that may be evaluated in this article, or claim that may be made by its manufacturer, is not guaranteed or endorsed by the publisher.
